# Establishing Pompe Disease Newborn Screening: The Role of Industry

**DOI:** 10.3390/ijns6030055

**Published:** 2020-07-05

**Authors:** Joan M. Keutzer

**Affiliations:** Sanofi Genzyme, Cambridge, MA 02142, USA; keutzer@verizon.net

**Keywords:** newborn screening, Pompe disease, acid α-glucosidase

## Abstract

When clinical trials for enzyme replacement therapy for Pompe disease commenced, a need for newborn screening (NBS) for Pompe disease was recognized. Two methods for NBS for Pompe disease by measuring acid α-glucosidase in dried blood spots on filter paper were developed in an international collaborative research effort led by Genzyme. Both methods were used successfully in NBS pilot programs to demonstrate the feasibility of NBS for Pompe disease. Since 2009, all babies born in Taiwan have been screened for Pompe disease. Pompe disease was added to the Recommended Uniform (Newborn) Screening Panel in the United States in 2015. NBS for Pompe disease is possible because of the unprecedented and selfless collaborations of countless international experts who shared their thoughts and data freely with the common goal of establishing NBS for Pompe disease expeditiously.

## 1. Introduction

We frequently hear that “it takes a village” to get things done. In the case of Pompe disease newborn screening (NBS), “it took the world”. NBS for Pompe disease resulted from an unprecedented international collaboration among patients with Pompe disease, the Pompe disease medical and scientific communities, government agencies, and industry. Starting in 1999, the baton was passed between research groups who acted urgently to generate the data required, develop methods for newborn screening for Pompe disease, and begin to establish newborn screening for Pompe disease.

In 1998, Genzyme (now Sanofi Genzyme) initiated a clinical development program for enzyme replacement therapy (ERT) for Pompe disease. During meetings to plan the clinical trials, physicians voiced concerns about the ability of patients with Pompe disease to get an early and accurate diagnosis. They cautioned that if the deficiencies in diagnostic testing were not addressed, that Genzyme would not be able to conduct successful clinical trials with ERT in Pompe disease because some symptoms would be irreversible. Thus, diagnostic delays could result in the inability to determine if ERT for Pompe disease was a viable treatment option. Patients with later-onset Pompe disease (LOPD) referred to the test as unreliable and invasive. Some patients told Genzyme that they had three muscle biopsies before they received a diagnosis. Parents of patients with infantile-onset Pompe disease (IOPD) mentioned waiting two grueling months after the collection of a skin biopsy to get a diagnosis. There was no assay that could use blood samples to diagnose Pompe disease.

In response, I assembled a team of Genzyme colleagues to determine if there was a role that we could play in improving diagnostics for Pompe disease. The team consisted of members from Research and Development, Genzyme Diagnostics, Genzyme Genetics, Regulatory Affairs, and the Pompe disease Clinical Development Team. The team interviewed multiple Pompe disease stakeholders, including patients, physicians, and diagnostic laboratories. Ultimately, Henri Termeer, the CEO of Genzyme, decided to sponsor international research to develop assays useful for NBS, diagnosis using blood samples, patient monitoring, and genotyping for Pompe disease. This decision was not supported unanimously internally. Some felt very strongly that it created a conflict of interest if a company was developing therapeutic and diagnostic testing for the same disease. Several healthy conversations about our role in patient diagnosis ensued and led to a commitment to keep our involvement in assay development scientific and make the resultant intellectual property and methodologies available to all. Much of the work was co-sponsored or sponsored by Genzyme. In 2003, Genzyme established an R&D lab to optimize, validate, and transfer methodology. The team was active through 2016.

## 2. Early Attempts to Develop Newborn Screening Assays

We hoped that NBS for Pompe disease could replicate what was done for other enzyme deficiencies, like PKU, and measure the accumulation of the enzyme’s substrate in dried bloodspots on filter paper (DBS) in a multiplex assay that could screen for multiple treatable lysosomal storage disorders (LSDs) simultaneously. In 2000, we started to collaborate with Sarah Young and David Millington on work they had initiated earlier. They developed the urine Hex4 assay, which is an indirect measure of elevated glycogen. The assay did not translate into an assay for DBS, but it is useful for monitoring patients with Pompe disease [1].

We knew that the measurement of acid α-glucosidase enzyme activity in mixed leukocytes and whole blood was complicated by the presence of another alpha-glucosidase, maltase glucoamylase (MGA), which is active at an acid pH and masks the deficiency of acid α-glucosidase; therefore, we sponsored the search for an inhibitor of MGA so that acid α-glucosidase could be quantitated in blood. For the first two years, no one found a useful inhibitor, but labs in Europe, Asia, and the United States kept trying. At International Conference on Inborn Errors of Metabolism (ICIEM) in Cambridge, UK in September 2000, Nestor Chamoles from the Laboratorio Chamoles in Buenos Aires, Argentina presented a poster that demonstrated that it was possible to measure the lysosomal enzyme alpha-L-iduronidase in DBS and differentiate DBS from patients with MPS I from DBS from obligate heterozygotes and healthy individuals [2]. We encouraged Chamoles to try to create an assay for acid α-glucosidase in DBS using 4-methylumbelliferyl-α-d-glucopyranoside (4-MUG) as the substrate.

## 3. Measuring Acid α-Glucosidase in Dried Bloodspots on Filter Paper

We knew that NBS was relying more heavily on multiplex assays, so we continued to search for technologies that could be used in a multiplex assay for LSDs. Our assumption was that like the other multiplex assays in NBS, the assay would measure analytes (substrates of the missing enzymes) in DBS, but that changed to wanting to measure several enzyme activities in a multiplex assay. At the same ICIEM meeting in Cambridge, UK in September 2000, Frantisek Turecek, C. Ronald Scott, and Michael H. Gelb from the University of Washington presented an assay that measured acid sphingomyelinase and β-galactocerebrosidase in skin fibroblast homogenates for clinical laboratory diagnosis [3]. The assay used novel biotinylated substrate conjugates, purification of the products on streptavidin agarose beads, and quantification of products using electrospray ionization mass spectrometry with stable-isotope-labeled internal standards that were chemically identical to the products of the enzymatic reactions. We invited them to join the efforts and their initial goal was to develop a multiplex assay for six lysosomal storage disorders Pompe disease, MPS I, Fabry disease, Gaucher disease, Krabbe disease, and Niemann–Pick disease types A and B.

In 2003, Gabriella Niizawa from the Chamoles lab developed a fluorometric assay using 4-methylumbelliferyl-α-d-glucopyranoside (4-MUG) as the substrate that could differentiate DBS from patients with IOPD from obligate heterozygotes and healthy individuals. The method used maltose to inhibit MGA since MGA has a higher affinity for maltose than acid α-glucosidase [4]. Chamoles invited X. Kate Zhang and me to his lab to run the assay and in June 2003, and allowed us to take his protocol into the Genzyme LSD assay development lab so we could try to optimize the protocol and adapt it from one using 1.5 mL microcentrifuge tubes to one that uses in 96-well plates and could be automated. Our activities were limited by the paucity of samples from patients previously diagnosed with Pompe disease.

In September 2003, Genzyme received a phone call from a father from Peru. He wanted to know how to access treatment for Pompe disease because his 4-month-old baby was suspected of having the disease because her brother died from Pompe disease. The baby did not have a confirmed diagnosis. We talked to Chamoles who said he was not ready to use the DBS assay for suspected cases but would test and report a result if Genzyme tested duplicate samples blinded and the results concurred. We requested duplicate DBS from the baby, the mother, the father, and someone unrelated. Peru did not have an NBS program, so we had to use a courier to deliver DBS cards to the clinic. Chamoles and Genzyme had similar results. Samples from both parents and the unrelated donor were in the normal range and the sample from the baby was consistent with a diagnosis of Pompe disease. The child and her family relocated to Durham, NC to participate in the ongoing clinical trial at Duke University Medical Center. The diagnosis of Pompe disease was confirmed at Duke using an assay in fibroblasts. This patient was the first identified with Pompe disease using the new blood-based assay and we gained confidence in the methodology.

Chamoles, Gelb, and the Genzyme R&D Team met at ICIEM in Brisbane in September 2003 where Chamoles presented data on maltose inhibition of MGA. We agreed to meet as soon as possible to discuss sharing lab methods. At the time, Gelb did not have a DBS assay for Pompe disease. In January 2004, Genzyme hosted a mini symposium to generate a collaborative research agenda. At that meeting, Gelb and Turecek revealed that they identified 80 µM acarbose (a drug used to control blood glucose levels in type 2 diabetes) to inhibit MGA [5]. However, all three labs had issues with sample availability. Chamoles had a limited number of samples because he collected DBS from patients undergoing diagnostic evaluation for Pompe disease and from their parents who were considered obligate carriers, but he shared them. Genzyme agreed that they would adapt both methods that used microcentrifuge tubes to 96-well dishes so that the relative effectiveness of maltose and acarbose could be studied more easily.

## 4. Optimizing the Methods

During the adaptation, optimization and validation activities led by Helmut Kallwass, the Genzyme R&D team, determined that acarbose was superior to maltose in the assay and that 8 µM acarbose was superior to 80 µM acarbose. Although 8 µM and 80 µM acarbose selectively blocked MGA over acid α-glucosidase, use of 8 uM acarbose inhibited less acid α-glucosidase (i.e., acarbose does have finite affinity for acid α-glucosidase but has higher affinity for MGA). However, we thought it was important to compare the DBS assay to the fibroblast assay in matched samples. Fortunately, in anticipation of evaluating the DBS assay, the R&D group at Duke University under the leadership of Deeksha Bali collected DBS from some IOPD patients for which they had fibroblast cultures. Bali’s team saw discrimination between patients with IOPD, obligate heterozygotes, and controls using 8 μM acarbose and the results using acarbose compared well with those using the skin fibroblast assay in the patients [6].

The Genzyme Clinical Development Team assisted in sample collection. In 2003, the informed consent and protocol for the pivotal Phase 3 clinical trial for patients with IOPD (A Study of the Safety and Efficacy of rhGAA in Patients with Infantile-onset Pompe Disease (NCT00059280)) were amended to include collecting DBS for developing DBS enzyme activity assays for acid α-glucosidase. In 2004, a Prospective, Observational Study in Patients with Late-Onset Pompe Disease (NCT00077662) included an optional request for DBS samples. In 2005, A Placebo-Controlled Study of Safety and Effectiveness of Myozyme (alglucosidase alfa) in Patients with Late-Onset Pompe Disease called the Late-Onset Treatment Study [LOTS] (NCT00158600) included an optional request for DBS samples during screening. There was virtually 100% patient participation in donating DBS in the three clinical trials. Without these samples, we could not have progressed in developing NBS or blood-based diagnostic testing for Pompe disease.

The DBS assay consistently differentiated the 24 previously diagnosed IOPD and 100 patients screened for LOTS from controls. Testing DBS samples from 61 patients from LOPOS previously diagnosed with LOPD revealed that 58 had results consistent with a diagnosis of Pompe disease and three had results in the normal range. Measuring acid α-glucosidase enzyme activity in fibroblasts and acid α-glucosidase gene (*GAA*) sequence analysis confirmed that the three patients with a neuromuscular disease were misdiagnosed with Pompe disease [7]. Four fibroblast samples had ≤1% residual enzyme activity so results from fibroblasts cannot be used to predict disease phenotype in cases identified by newborn screening. The results in the DBS assay were similar for IOPD and LOPD, so the DBS assay cannot be used to predict disease phenotype.

It is worth noting that all participating DBS collection sites were trained on how to make DBS via conference call and none of the DBS received from the clinical sites were unsatisfactory for use in assay development; this suggested that it might be feasible to use DBS samples for clinical diagnosis. DBS sampling permits access to testing for metabolic diseases in remote areas; DBS samples can be conveniently collected and shipped through the mail to a distant laboratory for analysis.

## 5. Early Experience with the Acid α-Glucosidase Enzyme Assay

Paul (Wuh-Liang) Hwu and Nancy (Yin-Hsiu) Chien invited Chamoles and I to a meeting at the Asian Regional International NBS Meeting in September 2004. They proposed that they were uniquely situated to conduct the first large-scale Pompe disease NBS pilot. They described a plan to treat those diagnosed with IOPD immediately and follow the patients’ outcomes using the protocols identical to the pivotal alglucosidase alfa clinical trial in IOPD, since they were a clinical trial site. We transferred that methodology from our Genzyme R&D lab to the National Taiwan University Hospital (NTUH) NBS Center in October 2004. NTUH introduced the word’s first Pompe disease NBS pilot program at the end of 2005 [8]. Yuan-Tsong Chen from Academia Sinica in Taipei, Taiwan collaborated in the design and execution of the program.

Initially, the NTUH lab ran three separate fluorometric assays to measure acid α-glucosidase, neutral α-glucosidase (NAG), and maltase-glucoamylase (MGA) activities. NAG and MGA were used to differentiate cases of Pompe disease from false positive cases. As the pilot progressed and the lab had more experience, cautious and methodical adjustments of assay cut-offs were used to minimize the risk of false positive and false negative test results. During the pilot, NTUH identified the high prevalence of the *GAA* pseudodeficiency allele (p.G576S) and the increase in phenotype severity when the polymorphism is in cis [9]. Since 2009, all newborns in Taiwan have been screened for Pompe disease. In 2015, NTUH adopted the tandem mass spectrometry-based multiplex LSD enzyme assay.

Today, it seems obvious that the DBS assay with acarbose is useful in diagnosing Pompe disease since all assays for Pompe disease using samples from blood include acarbose, but previous experiences with Pompe disease diagnoses and misdiagnoses left many skeptical of the new method in its early days. In December 2006, Genzyme sponsored a meeting of The Pompe Disease Diagnostic Working Group in London to establish a consensus regarding the application of these new assays for the laboratory diagnosis of Pompe disease. The Working Group consisted of scientists, geneticists, and clinicians working in the field of Pompe disease. The group agreed that cultured skin fibroblasts had been the gold standard, but was rapidly being replaced by assays in blood samples because they are less invasive, more rapid, and are easier to standardize [10]. The meeting was an important step in bringing laboratories and clinicians together to agree that the DBS assay using acarbose to inhibit MGA was useful to get a presumptive positive diagnoses for Pompe disease and that at least one secondary positive test (for example, *GAA* sequence analysis) would support a biochemical diagnosis of Pompe disease. Genzyme’s R&D team made the DBS assay protocol available to anyone who requested it and trained labs at Genzyme and remotely as requested. This hastened the adoption of the fluorescent assay to measure acid α-glucosidase enzyme activity and increased the number of patients being diagnosed with Pompe disease worldwide.

While the team at NTUH was using the Genzyme R&D adaptation of the Chamoles lab assay in their NBS program, the Genzyme R&D team that was led by Zhang was adapting the tandem mass spectrometry-based assay for multiple LSD enzymes from the University of Washington [5] to a high-throughput method. However, Genzyme did not have any experience in NBS, so we asked each NBS lab in the United States to answer a survey about the published assay and its suitability for use in NBS. For each survey returned, Genzyme made a donation to the Association of Public Health Laboratories. Based on the responses from NBS labs, Genzyme transformed the assay into a robust high throughput 96-well plate format assay using a robotic liquid handling system for sample transfer and minimizing the detergent burden on the mass spectrometer by replacing Triton X-100 with CHAPS. We addressed the environmental concerns of the NBS labs by replacing chloroform with ethyl acetate [11]. We created a reliable supply of the substrates and internal standards manufactured in our GMP facility using validated manufacturing processes.

We facilitated the use of the assay in NBS through a ten year (2006–2016) donation and grant agreement with the Centers for Disease Control and Prevention (CDC) Foundation so that (1) the reagents could be distributed without charge to NBS labs before they were commercially available, (2) NBS labs could be trained on lysosomal storage disorder NBS methods, and (3) quality control and external quality assurance samples were available for use in NBS [12]. The reagents were manufactured at Genzyme Pharmaceuticals in Liestal, Switzerland and tested for quality and distributed to the CDC by Genzyme Diagnostics in Framingham, Massachusetts.

## 6. Pompe Disease and the Recommended Uniform (Newborn) Screening Panel

In May 2006, Priya Kishnani nominated Pompe disease for inclusion in the Recommended Uniform (Newborn) Screening Panel (RUSP) in the United States. In October 2008, the nomination was denied. The Advisory Committee recommended additional studies before the condition could be re-nominated. The response to the nomination stated that “the initial test specificity for Pompe disease alone should be improved in comparison to the data shown by the Taiwan study, further evaluation of alternative screenings methods that could be multiplexed to target additional conditions (for example, other Lysosomal Storage Disease conditions) in order to decrease the burden on public health laboratories, and a standardized method of diagnosis after a positive newborn screen is required [13].

The Pompe disease community and Genzyme were disappointed by the response, but Genzyme remained staunch and committed to two additional efforts: funding an NBS study in Washington state and sponsoring an expert group to generate guidance for NBS for Pompe disease for practitioners around the world.

Fortuitously, the team in Washington had just developed a true triplex enzyme assay for Pompe disease, Fabry disease, and MPS I in which the enzymatic activities of acid α-glucosidase, α-galactosidase A, and α-l-iduronidase were quantified in DBS using a single assay buffer [14]. The validated triplex assay was transferred expeditiously to the Washington State NBS Lab and used to measure enzyme activities in approximately 110,000 DBS that had been stored at 18 °C for 8–10 months after collection. The word approximately is used because the study started before the reagents for Fabry disease and MPS I were ready for distribution by the CDC and results were not reported for tests done using research grade reagents, so the number of results reported vary by disease [15]. The study was anonymous but confirmed the diagnosis by DNA sequencing using a duplicate punch from the same sample. The positive predictive values for Pompe disease, Fabry disease, and MPS I were 0.24, 0.43, and 0.33, respectively. The false positive rates were 1/8600, 1/12,100, and 1/17,500, respectively.

The Washington State NBS data generated a lot of hope in the MPS I and Pompe disease communities. In late 2011, cross-functional teams of patient advocacy groups, diagnostic experts, disease experts, and NBS experts were established for MPS I and Pompe disease. MPS I and Pompe disease were nominated for inclusion in the RUSP in 2012; and the nominations were approved for formal data review. Evidence review for Pompe disease preceded evidence review for MPS I because the Advisory Committee budget could not support parallel evidence reviews. In 2013, the Advisory Committee recommend adding Pompe Disease to the RUSP. In 2015, Pompe disease was added to the RUSP [16]. In 2015, the Advisory Committee recommended adding MPS I to the RUSP. In 2016, MPS I was added to the RUSP [16].

Recognizing the importance of consistency in NBS, Sanofi Genzyme facilitated and provided financial support for the meeting of the Pompe Disease NBS Working Group, which was led by Hwu and Kishnani to discuss and develop a general guidance document for NBS for Pompe disease for practitioners around the world. The guidance was published in a supplement in Pediatrics [17]. Sub-teams from the Working Group authored guidelines for NBS for Pompe Disease; the initial evaluation of patients after positive NBS and recommended algorithms leading to a confirmed diagnosis of Pompe disease; management of confirmed newborn-screened patients with Pompe disease across the disease spectrum; and the role of genetic counseling in Pompe disease after patients are identified through NBS.

NBS for Pompe disease is possible because of the unprecedented and selfless collaborations of countless international experts who shared their thoughts and data without delay so that the next step in the process could be initiated during the lag required for preparation of publications. NBS for Pompe disease was facilitated by the financial, organizational, and technical contributions from Genzyme and Sanofi Genzyme.
